# Sentinel Surveillance of HIV-1 Transmitted Drug Resistance, Acute Infection and Recent Infection

**DOI:** 10.1371/journal.pone.0025281

**Published:** 2011-10-06

**Authors:** Hong-Ha M. Truong, Timothy A. Kellogg, Willi McFarland, Brian Louie, Jeffrey D. Klausner, Susan S. Philip, Robert M. Grant

**Affiliations:** 1 Department of Medicine, University of California San Francisco, San Francisco, California, United States of America; 2 Gladstone Institute of Virology and Immunology, San Francisco, California, United States of America; 3 Department of Public Health, San Francisco, California, United States of America; McGill University, Canada

## Abstract

**Background:**

HIV-1 acute infection, recent infection and transmitted drug resistance screening was integrated into voluntary HIV counseling and testing (VCT) services to enhance the existing surveillance program in San Francisco. This study describes newly-diagnosed HIV cases and characterizes correlates associated with infection.

**Methodology/Principal Findings:**

A consecutive sample of persons presenting for HIV VCT at the municipal sexually transmitted infections (STI) clinic from 2004 to 2006 (N = 9,868) were evaluated by standard enzyme-linked immunoassays (EIA). HIV antibody-positive specimens were characterized as recent infections using a less-sensitive EIA. HIV-RNA pooled testing was performed on HIV antibody-negative specimens to identify acute infections. HIV antibody-positive and acute infection specimens were evaluated for drug resistance by sequence analysis. Multivariable logistic regression was performed to evaluate associations. The 380 newly-diagnosed HIV cases included 29 acute infections, 128 recent infections, and 47 drug-resistant cases, with no significant increases or decreases in prevalence over the three years studied. HIV-1 transmitted drug resistance prevalence was 11.0% in 2004, 13.4% in 2005 and 14.9% in 2006 (p = 0.36). Resistance to non-nucleoside reverse transcriptase inhibitors (NNRTI) was the most common pattern detected, present in 28 cases of resistance (59.6%). Among MSM, recent infection was associated with amphetamine use (AOR = 2.67; p<0.001), unprotected anal intercourse (AOR = 2.27; p<0.001), sex with a known HIV-infected partner (AOR = 1.64; p = 0.02), and history of gonorrhea (AOR = 1.62; p = 0.03).

**Conclusions:**

New HIV diagnoses, recent infections, acute infections and transmitted drug resistance prevalence remained stable between 2004 and 2006. Resistance to NNRTI comprised more than half of the drug-resistant cases, a worrisome finding given its role as the backbone of first-line antiretroviral therapy in San Francisco as well as worldwide. The integration of HIV-1 drug resistance, recent infection, and acute infection testing should be considered for existing HIV/STI surveillance and prevention activities, particularly in an era of enhanced efforts for early diagnosis and treatment.

## Introduction

HIV-1 acute infection, recent infection and antiretroviral (ARV) drug resistance are of clinical and public health significance. Acute infection is defined as the time interval between the acquisition of HIV infection and seroconversion. The high viral load during this stage of infection increases biological transmissibility [1], [2]. Risk of transmission during acute infection is 8–22 times greater on a per-act basis than later stages of infection [2], [3]. In addition, most persons with acute infection are unaware of their status and may engage in risky behaviors that enable further transmission [2], [4]. Identification of persons with recent infections after the acute period, e.g., through laboratory-based testing algorithms, may help track the leading edge of the HIV epidemic within the community by distinguishing newly-transmitted infections from newly-diagnosed but long-term infections [5]. The transmission of drug-resistant strains has been documented, which can potentially impact virologic, immunologic and broader health outcomes by decreasing the effectiveness of antiretrovirals [6]–[9].

Sexually transmitted infections (STI) clinic patients are a sentinel population used for HIV surveillance worldwide based on the rationale that high numbers of persons newly infected with HIV may first present at such facilities since the behaviors that place individuals at risk for acquiring STI and HIV are similar [10]. The manifestly high risk for HIV among STI clinic patients also argues in favor of screening for recent infection among persons who are HIV-RNA positive/antibody-positive (RNA+/Ab+) and for acute infection among persons who are HIV-RNA positive/antibody-negative (RNA+/Ab−). HIV-1 drug resistance testing can also be integrated into the screening algorithm at STI clinics to enhance existing surveillance efforts. From 2004 to 2006, testing for HIV acute infection and transmitted drug resistance was conducted at the voluntary counseling and testing (VCT) program of San Francisco's sole municipal STI clinic. This study describes the prevalence of HIV-1 transmitted drug resistance, acute infection, and recent infection and characterizes their associated correlates.

## Methods

### Ethics Statement

The study received approval from the Institutional Review Board at the University of California, San Francisco. No additional data were collected for this public health surveillance activity; therefore, written patient consent was not required.

### Study Population

A consecutive sample of persons presenting for confidential HIV VCT at the San Francisco municipal STI clinic from January 2004 to December 2006 (N = 9,868) were evaluated. Newly-identified HIV cases were considered to be ARV treatment-naïve since the study population was comprised of testers seeking to know their HIV status.

### Testing Algorithm

Specimens were screened using standard enzyme-linked immunoassays (EIA) (Vironostika HIV-1 Microelisa, bioMérieux, Durham, NC) and OraQuick Rapid Test (OraQuick Advance HIV 1/2 Antibody Test, Bethlehem, PA). Antibody-positive samples were confirmed using immunofluoresence assays (Fluorognost HIV-1 IFA, Sanochemia Pharmazeutika AG, Neufeld, Vienna, Austria). HIV Ab+ specimens were characterized as recent HIV infections using a testing approach referred to as “STARHS”, which stands for “Serological Testing Algorithm for Recent HIV Seroconversion”. STARHS distinguishes recent from long-term infections using two EIA: a standard assay (Vironostika HIV-1) that is sensitive to low levels of HIV antibody and a less-sensitive one (Vironostika-LS) that classifies recent infection using a 170 day window period [95% CI: 145, 200 days] and an optical density cut-off value of 1.0 [5]. HIV Ab- specimens were screened using a quantitative HIV-1 RNA assay with an analytic sensitivity down to 75 copies/ml (Versant HIV 3.0, Bayer Diagnostics, Emeryville, CA). Initially, a two-stage pooling strategy was applied, with a 50 specimen master pool and 10 specimen intermediate pools. A revised strategy with 10 specimens in each master pool, followed by individual testing of specimens in any positive pools was used to expedite turn-around time for results.

Newly-detected HIV infections (RNA+/Ab+ and RNA+/Ab−) were evaluated for drug resistance by viral genotype population sequencing (TRUGENE HIV-1 Genotyping Kit, Bayer Diagnostics, Emeryville, CA). The assay detects mutations in the protease and reverse transcriptase sequences of the HIV-1 genome that confer resistance to ARVs. Viral genotypic sequencing was performed on 370 HIV+ specimens and interpretable sequences were generated for 348 specimens, yielding a 94% assay success rate. Sequencing results were interpreted using guidelines from the manufacturer (Version 12 Rules), IAS-USA, and the Stanford University HIV-1 Drug Resistance Surveillance Program [11], [12].

### Data Collection

Demographic characteristics and risk behavior information were obtained from standardized intake data collection forms that were administered by test counselors in private settings as a routine part of HIV VCT services. Correlates of acute and recent HIV infection and transmitted drug resistance were based on secondary analysis of existing data. Data available for analysis included demographic characteristics (e.g., gender, age, racial/ethnic identification, sexual orientation), sexual behavior (e.g., gender of sex partners, number of sex partners, engaging in unprotected sexual intercourse, and sex with a known HIV-infected partner within the past twelve months), substance use (e.g., use of injection drugs, non-injection drugs, and alcohol within the past twelve months), and medical history (e.g., perceived HIV status before the current test, number of previous HIV tests, and STD history within the past two years).

### Analysis

HIV infection, recent infection, acute infection, and drug resistance were compared across years. HIV infection cases included all persons who were either antibody positive by standard antibody testing or who were acutely infected. Stages of HIV infection were defined as follows: (i) acute infections were RNA+/Ab−, (ii) recent infections were RNA+/Ab+ and transmission likely occurred within the past 170 days, and (iii) long-term infections were RNA+/Ab+ and transmission likely occurred beyond the past 170 days. HIV-1 drug resistance was defined as having a single major mutation or several minor mutations known to confer intermediate or high levels of resistance to ARVs. HIV-1 incidence estimates were calculated by dividing the number of persons with recent infection by persons at risk (recently infected plus uninfected) and annualized using the following formula: crude incidence x [(365 days/170 days) x 100%] [4]. For the analysis, cases of HIV infection, recent infection, and acute infection were compared to HIV-negative cases. Only HIV-infected persons were included in the analysis to assess the predictors of drug resistance. Logistic regression models were fitted to identify independent predictor variables for the HIV outcomes of recent infection, acute infection, and drug resistance and to adjust for confounding factors. Candidate variables were first evaluated by bivariate analysis. Initial variable selection for the multivariate model was based on a p-value cut-off of 0.20 for inclusion. Backward elimination was used for final model selection using a p-value of 0.10 for retention. Ninety-five percent confidence intervals computed on the odds ratios were derived from the coefficients and their respective standard errors. Temporal trends were assessed using the Cochran-Armitage test.

## Results

The HIV VCT population consisted of 3,789 testers in 2004, 2,921 testers in 2005, and 3,158 testers in 2006. Testers were 84.4% male, 14.1% female, and 1.3% transgender. By age, 18.1% were under 25 years old, 41.1% were 25–34, 27.6% were 35–44, and 13.0% were 45 and older. The testing population was comprised of 54.4% whites, 20.0% Latinos, 10.6% African-Americans, 11.8% Asian/Pacific Islanders, and 3.0% other/mixed race. Risk exposure data were available for 93.8% of testers (n = 9,380), of which 73.1% were men who have sex with men (MSM), 23.6% were heterosexual, 1.9% were injection drug users (IDU), and 1.4% were transgender. Among all testers, 36.2% reported unprotected anal sex (UAI), 16.9% had sex with a known HIV-positive partner, 11.7% used amphetamines, 4.1% used injection drugs, 14.7% had a history of gonorrhea, 9.5% had a history Chlamydia, and 3.2% had a history of syphilis.

There were 380 HIV infections newly diagnosed between 2004 and 2006. Newly-identified HIV cases comprised of 223 long-term infections (58.7%), 128 recent infections (33.7%), and 29 acute infections (7.6%). Eleven of the 29 acute infection specimens were originally screened by the rapid test. Table 1 presents HIV cases stratified by demographic characteristics, risk exposure categories, and predictors of risk. HIV prevalence was highest among men (4.34%), 35–44 year olds (4.78%), African-Americans (4.89%), and MSM (4.78%), with regards to the proportion of cases relative to testers with the same demographic characteristics and risk exposure categories. Acute infections were mainly detected in testers who were MSM (93.1%), white (44.8%), and 35–44 year old (34.2%). Recent infection cases were primarily testers who were MSM (85.9%), white (53.9%), and 25–34 year old (37.5%).

**Table 1:** HIV infections stratified by demographic characteristics, risk exposure categories and predictors of risk, San Francisco, 2004–2006.

	All Testers	HIV-Negative		All HIV Infection			Long-Term Infection		Recent Infection			Acute Infection		HIV-1 Drug Resistance		
	N = 9,868	N = 9,488		N = 380			N = 223		N = 128			N = 29		Genotyped N = 362	Drug-Resistant Cases N = 47	
		n	% 1	n	% 2	HIV Prevalence % [95% CI]^3^	n	% 4	n	% 5	HIV Incidence % [95% CI]	n	% 6	n	n	% 7
**Year**																
2004	3,789	3,653	38.5	136	35.8	3.59 [3.48, 4.25]	81	36.3	44	34.4	2.55 [1.81, 3.35]	11	37.9	136	15	31.9
2005	2,921	2,794	29.4	127	33.4	4.35 [3.64, 5.15]	73	32.7	44	34.4	3.32 [2.35, 4.34]	10	34.5	112	15	31.9
2006	3,158	3,041	32.1	117	30.8	3.70 [3.07, 4.42]	69	30.9	40	31.3	2.78 [1.94, 3.71]	8	27.6	114	17	36.2
**Gender**																
Men	8,332	7,970	84.0	362	95.3	4.34 [3.92, 4.80]	210	94.2	125	97.7	3.31 [2.68, 3.84]	27	93.1	345	41	87.2
Women	1,396	1,387	14.6	9	2.4	0.64 [0.30, 1.22]	7	3.1	2	1.6	0.31 [0.04, 1.11]	0	0.0	8	2	4.3
Transgender	128	123	1.3	5	1.3	3.91 [1.28, 8.88]	4	1.8	1	0.8	1.73 [0.04, 9.35]	0	0.0	5	1	2.1
Missing	12	8	0.1	4	1.1	33.33 [9.92, 65.11]	2	0.9	0	0.0	0.00 [0.00, 66.05]	2	6.9	4	3	6.4
**Age**																
Under 25	1,789	1,742	18.4	47	12.4	2.63 [1.94, 3.48]	17	7.6	25	19.5	3.03 [1.94, 4.43]	5	17.2	45	5	10.6
25–34	4,059	3,919	41.3	140	36.8	3.45 [2.91, 4.06]	83	37.2	48	37.5	2.59 [1.87, 3.37]	9	31.0	137	16	34.0
35–44	2,719	2,589	27.3	130	34.2	4.78 [4.01, 5.65]	80	35.9	40	31.3	3.26 [2.26, 4.30]	10	34.5	124	17	36.2
45 and older	1,284	1,226	12.9	58	15.3	4.52 [3.45, 5.80]	40	17.9	15	11.7	2.59 [1.40, 4.14]	3	10.3	51	6	12.8
Missing	17	12	0.1	5	1.3	29.41 [10.31, 55.96]	3	1.3	0	0.0	0.00 [0.00, 46.62]	2	6.9	5	3	6.4
**Race/Ethnicity**																
White	5,359	5,181	54.6	178	46.8	3.32 [2.86, 3.84]	96	43.0	69	53.9	2.82 [2.15, 3.50]	13	44.8	173	20	42.6
Latino	1,968	1,874	19.8	94	24.7	4.78 [3.88, 5.81]	57	25.6	30	23.4	3.37 [2.21, 4.68]	7	24.1	91	10	21.3
African-American	1,042	991	10.4	51	13.4	4.89 [3.67, 6.39]	29	13.0	19	14.8	4.03 [2.36, 6.12]	3	10.3	49	8	17.0
Asian/Pacific Islander	1,162	1,138	12.0	24	6.3	2.07 [1.33, 3.06]	16	7.2	6	4.7	1.12 [0.41, 2.41]	2	6.9	22	4	8.5
Other/Mixed	293	270	2.8	23	6.1	7.85 [5.04, 11.55]	17	7.6	4	3.1	3.11 [0.80, 7.51]	2	6.9	19	1	2.1
Missing	44	34	0.4	10	2.6	22.73 [11.47, 37.84]	8	3.6	0	0.0	0.00 [0.00, 18.01]	2	6.9	8	4	8.5
**Risk Exposure**																
MSM	6,859	6,531	68.8	328	86.3	4.78 [4.29, 5.31]	191	85.7	110	85.9	3.54 [2.83, 4.15]	27	93.1	315	36	76.6
Heterosexual	2,212	2,196	23.1	16	4.2	0.72 [0.41, 1.17]	11	4.9	5	3.9	0.49 [0.16, 1.13]	0	0.0	15	5	10.6
IDU (non-MSM)	177	172	1.8	5	1.3	2.82 [0.92, 6.47]	1	0.4	2	1.6	2.44 [0.29, 8.77]	0	0.0	3	0	0.0
Transgender	132	129	1.4	3	0.8	2.27 [0.47, 6.50]	2	0.9	1	0.8	1.65 [0.04, 9.07]	0	0.0	3	1	2.1
Missing	488	458	4.8	30	7.9	6.15 [4.19, 8.66]	18	8.1	10	7.8	4.57 [2.11, 8.10]	2	6.9	26	5	10.6
**Predictors of Risk**																
Sex with Known HIV+ Partner	1,668	1,532	16.1	136	35.8	8.15 [6.88, 9.57]	75	33.6	53	41.4	7.15 [5.11, 8.93]	8	27.6	114	13	27.7
Unprotected Anal Intercourse	3,571	3,335	35.1	236	62.1	6.61 [5.82, 7.47]	135	60.5	88	68.8	5.50 [4.25, 6.52]	13	44.8	222	27	57.4
Amphetamine Use	1,156	1,045	11.0	111	29.2	9.60 [7.96, 11.45]	62	27.8	43	33.6	8.44 [5.78, 10.77]	6	20.7	102	12	25.5
Injection Drug Use	408	390	4.1	18	4.7	4.41 [2.64, 6.88]	8	3.6	10	7.8	5.37 [2.53, 9.68]	0	0.0	16	2	4.3
Syphilis	312	273	2.9	39	10.3	12.50 [9.04, 16.69]	27	12.1	8	6.3	6.03 [2.38, 10.86]	4	13.8	33	3	6.4
Gonorrhea	1,452	1,358	14.3	94	24.7	6.47 [5.26, 7.86]	52	23.3	33	25.8	5.06 [3.36, 6.86]	9	31.0	85	13	27.7
Chlamydia	933	892	9.4	41	10.8	4.39 [3.20, 4.82]	20	9.0	18	14.1	4.24 [2.46, 6.55]	3	10.3	40	6	12.8

Denominators:

1 HIV-Negative.

2 All HIV Infection.

3 All Testers.

4 Long-Term Infection.

5 Recent Infection.

6 Acute Infection.

7 Drug-Resistant Cases.

Prevalence of HIV-1 transmitted drug resistance was 13.0% overall during the three year period. A total of 47 drug-resistant cases were identified, 77% of which were detected in MSM. Table 2 shows the distribution of cases by drug class. Mutations conferring resistance to non-nucleoside reverse transcriptase inhibitors (NNRTI) were the most common pattern observed. NNRTI resistance was present in 28 of 47 cases (59.6%), nucleoside reverse transcriptase inhibitors (NRTI) resistance in 14 cases (29.8%), and protease inhibitors (PI) resistance in 14 cases (29.8%). There were 5 cases of dual-class resistance (2 NRTI/NNRTI cases, 2 NNRTI/PI cases, 1 NRTI/PI case) and 2 cases of multi-class resistance (NRTI/NNRTI/PI). The most commonly detected mutations conferring resistance to NRTI were M184V (10.6%), K219Q (10.6%), D67N (8.5%), L210W (8.5%), and M41L (8.5%). The most common resistant mutations to NNRTI were K103N (36.2%), Y181C (8.5%), and V108I (8.5%) and to PI were L90M (14.9%), V77I (10.6%), and D30N (6.4%).

**Table 2:** Distribution of HIV-1 drug-resistant cases by drug class, San Francisco, 2004–2006.

	2004		2005		2006	
	n	%	n	%	n	%
**Any Drug Class**	15	100	15	100	17	100
**NRTI**	5	33.3	4	26.7	5	29.4
**NNRTI**	11	73.3	8	53.3	9	52.9
**PI**	3	20.0	3	20.0	8	47.1
**Dual-Class**	3	20.0	1	6.7	1	5.9
**Multi-Class**	0	0.0	0	0.0	2	11.8

NRTI = nucleoside reverse transcriptase inhibitors.

NNRTI = non-nucleoside reverse transcriptase inhibitors.

PI = protease inhibitors.

The characteristics of HIV cases are presented in Table 1. Unprotected anal intercourse (UAI) in the past 12 months was reported by 60.5% of long-term infections, 68.8% of recent infections, 44.8% of acute infections, and 57.4% of ARV drug-resistant cases. Sex with a known HIV-infected partner was reported by 33.6% of long-term infections, 41.4% of recent infections, 27.6% of acute infections and 27.7% of ARV drug-resistant cases. Amphetamine use was reported by 27.8% of long-term infections, 41.4% of recent infections, 27.6% of acute infections, and 25.5% of ARV drug-resistant cases. Injection drug use was reported by 3.6% of long-term infections, 7.8% of recent infections, and 4.3% of ARV drug-resistant cases. A history of gonorrhea was reported by 23.3% of long-term infections, 25.8% of recent infections, 31.0% of acute infections, and 27.7% of ARV drug-resistant cases.

**Figure 1:**
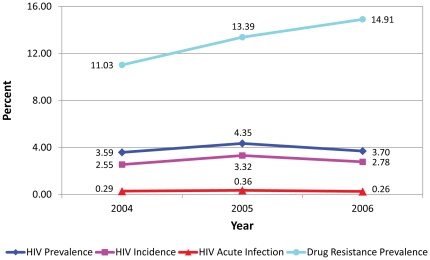
Temporal trends of HIV cases, San Francisco, 2004–2006.

**Figure 2:**
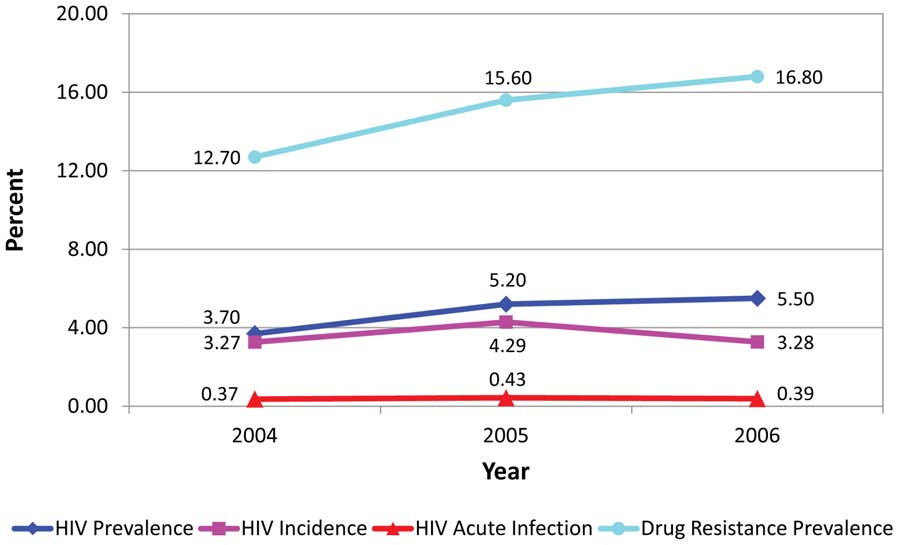
Temporal trends of HIV cases among men who have sex with men, San Francisco, 2004–2006.

There were no significant temporal trends among all testers in the prevalence of HIV infection (p = 0.74), acute infection (p = 0.80), and HIV incidence (p = 0.66), as shown in Figure 1. When the analyses were limited to MSM, there was a significant temporal trend in the prevalence of HIV infection (p = 0.01) but not for acute infection (p = 0.90) and HIV incidence (p = 0.94), as presented in Figure 2. Reported UAI increased significantly, from 35% in 2004 to 39% in 2006 among all testers (p = 0.002) and from 41% in 2004 to 46% in 2006 among MSM (p<0.001).

Prevalence of HIV-1 transmitted drug resistance did not decrease over the three year period among all testers (p = 0.36) and MSM (p = 0.39), as shown in Figures 1 and 2. There was an increase in the proportion of cases with PI resistance (p = 0.05) and a borderline increase in multi-class resistance (p = 0.07); however, there was no trend in NNRTI resistance (p = 0.94) and dual-class resistance (p = 0.36), as shown in Table 2.

Predictors of elevated risk for recent infection for all testers included male gender (AOR = 5.86 [95% CI: 1.84, 18.62]; p = 0.003), amphetamine use (AOR = 2.50 [95% CI: 1.70, 3.69]; p<0.001), sex with a known HIV-positive partner (AOR = 2.12 [95% CI: 1.47, 3.06]; p<0.001), UAI (AOR = 2.01 [95% CI: 1.40, 2.92]; p<0.001), African-American race/ethnicity (AOR = 1.88 [95% CI: 1.16, 3.05]; p = 0.01), and history of gonorrhea (AOR = 1.45 [95% CI: 0.97, 2.19]; p = 0.07). Asian/Pacific Islander race/ethnicity was protective for recent infection (AOR = 0.40 [95% CI: 0.17, 0.91]; p = 0.02). There were no significant correlates of acute infection or drug resistance.

Separate analyses were conducted for MSM, a risk group that accounted for 86.3% of HIV infection cases diagnosed. When analyses were restricted to MSM, recent infection was associated with amphetamine use (AOR = 2.67; p<0.0001), UAI (AOR = 2.27; p = 0.0002), sex with a known HIV-infected partner (AOR = 1.64; p = 0.02), and history of gonorrhea (AOR = 1.62; p = 0.03). Asian/Pacific Islander race/ethnicity (AOR = 0.41; p = 0.03) was associated with a lower risk for recent infection, while sex with an HIV-infected partner (AOR = 0.38; p = 0.03) was associated with a lower risk for drug resistance. Bivariate and multivariate risk models for MSM are detailed in Table 3.

**Table 3:** Bivariate and multivariate risk analyses of HIV recent infection, acute infection and drug resistance, MSM testers, San Francisco, 2004–2006.

Predictors of Risk	Recent HIV Infection						Acute HIV Infection						HIV-1 Drug Resistance					
	OR	95% CI	P	AOR	95% CI	P	OR	95% CI	P	AOR	95% CI	P	OR	95% CI	P	AOR	95% CI	P
**Demographics**																		
Year	0.84	0.67, 1.05	0.13				0.89	0.53, 1.49	0.65				0.98	0.65, 1.49	0.93			
Age (10 year increase)	0.93	0.76, 1.12	0.44				1.04	0.64, 1.69	0.88				1.08	0.73, 1.59	0.71			
**Race/Ethnicity**																		
White	Reference						Reference						Reference					
Latino	1.33	0.85, 2.07	.011				1.30	0.48, 3.50	0.60				0.97	0.41, 2.30	0.95			
African-American	1.67	0.89, 3.12	0.21				1.14	0.30, 4.35	0.84				1.15	0.37, 3.12	0.80			
Asian/Pacific Islander	0.44	0.19, 1.03	0.06	0.41	0.18, 0.93	0.03	0.57	0.70, 4.67	0.60				1.07	0.29, 3.98	0.92			
Other/Mixed	1.52	0.54, 4.21	0.43				--	--	--				0.65	0.08, 5.33	0.68			
**Behavioral/Biological Indicators**																		
Unprotected Anal Intercourse	3.15	2.01, 4.93	<0.0001	2.27	1.47, 3.52	<0.001	0.42	0.17, 1.03	0.06				1.67	0.91, 3.05	0.10			
Sex with Known HIV+ Partner	2.40	1.62, 3.52	<0.0001	1.64	1.10, 2.47	0.02	1.02	0.41, 2.52	0.96				0.49	0.21, 1.12	0.09	0.38	0.16, 0.91	0.03
Amphetamine Use	3.90	2.60, 5.80	<0.0001	2.67	1.77, 4.04	<0.001	0.73	0.28, 1.94	0.52				0.55	0.24, 1.27	0.16			
Injection Drug Use	2.41	1.16, 5.02	0.02				--	--	--				0.58	0.07, 4.61	0.61			
Gonorrhea	2.05	1.36, 3.10	0.0007	1.62	1.06, 2.47	0.03	1.86	0.76, 4.55	0.17	2.26	0.89, 5.73	0.08	1.61	0.77, 3.37	0.21	2.17	0.99, 4.77	0.05
Chlamydia	1.71	1.03, 2.86	0.04				0.99	0.28, 3.50	0.98				1.31	0.56, 3.39	0.58			
Syphilis	1.54	0.67, 3.55	0.31				1.86	0.59, 5.88	0.29				0.71	0.21, 2.47	0.59			

OR = Odds Ratio.

AOR = Adjusted Odds Ratio.

CI = Confidence Intervals.

P = P-value.

## Discussion

New HIV diagnoses, recent infections, acute infections, and transmitted drug resistance prevalence remained stable between 2004 and 2006. Slightly more than half of newly-diagnosed HIV cases were long-term infections, one-third were recent infections and close to one-tenth were acute infections. Nearly all acute and recent infection cases were detected in MSM. This result is most likely a reflection of high levels of repeat HIV testing in this population. A recent survey found 97% of MSM in San Francisco had ever tested and 34% had tested in the last 6 months [13]. Some studies have suggested that transmission by acutely-infected cases may account for 25–50% of recently-acquired infections [14], [15]. Testing for acute infection enhanced case detection by 7.6% and enabled referral for early care and potentially decreased risk of secondary transmission as a result of awareness of one's acute infection status.

Prevalence of transmitted HIV-1 drug resistance did not decrease over the three years studied. The majority of drug-resistant cases were detected in MSM and more than one-third of the cases reported having an STI within the past two years. In 85% of the resistant cases, resistance was limited to a single drug class. Resistance to NNRTI was the most common pattern observed, comprising more than half of the cases. The high proportion of cases with resistance to NNRTI is comparable to the national pattern reported by the U.S. Centers for Disease Control and Prevention [16]. The finding that a high proportion of resistant cases were associated with NNRTI mutations suggests that these mutations may be more common in source partners or more fit for transmission than other forms of drug-resistant HIV-1. The lack of a decrease in NNRTI resistance is particularly worrisome given that this drug class is the backbone of first-line antiretroviral therapy in San Francisco as well as worldwide, according to the recommendations of the most recent treatment guidelines by the U.S. Department of Health and Human Services and the International AIDS Society [17], [18].

The results from this study may not be generalizable to other STI clinics. The patient population characteristics may differ at other clinics, as MSM comprise a large percentage of testers at the San Francisco municipal STI clinic. However, the testing algorithm presented here can be implemented by clinics and public health departments to enhance HIV surveillance programs. The strategy of screening for acute HIV infections at STD clinics has been implemented in a number of U.S. cities. The proportion of acute infections detected in our study (7.6%) was comparable to the findings from STD clinics in New York City (8.6%) and higher than in Baltimore (1.3%) [19], [20]. In addition, nearly all of the acute HIV infection cases detected in New York City were among MSM, a result similar to our findings.

Our study illustrates how the integration of HIV-1 drug resistance testing with recent and acute infection screening can inform existing HIV/STI surveillance and prevention efforts. Knowledge of transmitted drug resistance prevalence and correlates of acute and recent infections can help target care and prevention strategies. Monitoring HIV-1 drug resistance prevalence in STI patients may be helpful for determining appropriate treatment and post-exposure prophylaxis regimens that are active against viruses circulating in the community.
