# Correction: Sentinel Surveillance of HIV-1 Transmitted Drug Resistance, Acute Infection and Recent Infection

**DOI:** 10.1371/annotation/69eda582-222c-4993-99a5-733db2dac07c

**Published:** 2011-11-01

**Authors:** Hong-Ha M. Truong, Timothy A. Kellogg, Willi McFarland, Brian Louie, Jeffrey D. Klausner, Susan S. Philip, Robert M. Grant

The first author's name contains an error in the citation. The correct citation is: Truong HM, Kellogg TA, McFarland W, Louie B, Klausner JD, et al. (2011) Sentinel Surveillance of HIV-1 Transmitted Drug Resistance, Acute Infection and Recent Infection. PLoS ONE 6(10): e25281. doi:10.1371/journal.pone.0025281 

